# A Development of New Material for 4D Printing and the Material Properties Comparison between the Conventional and Stereolithography Polymerised NVCL Hydrogels

**DOI:** 10.3390/jfb13040262

**Published:** 2022-11-22

**Authors:** Shuo Zhuo, Luke M. Geever, Elaine Halligan, Billy Shu Hieng Tie, Colette Breheny

**Affiliations:** 1 Material Research Institute, Technological University of the Shannon, Midlands Midwest, Dublin Road, N37 HD68 Athlone, Country Westmeath, Ireland; 2 Applied Polymer Technologies Gateway, Material Research Institute, Technological University of the Shannon, Midlands Midwest, Dublin Road, N37 HD68 Athlone, Country Westmeath, Ireland

**Keywords:** 4D printing, NVCL, material properties

## Abstract

The term 4D printing refers to the idea that the shape or properties of a printed object can be changed when an external stimulus is applied. In this contribution, a temperature-responsive polymer Poly (N-vinyl caprolactam) (PNVCL), which is normally prepared via radical free polymerization, was used to justify the 4D printing concept. As a result, by using a Stereolithography (SLA) 3D printer, 4D prints were successfully prepared. These prints were able to demonstrate intelligent and reversible expansion/shrinkage behaviour as the temperature increases and decreases. Additionally, in order to examine the differences in chemical structure, thermal properties, mechanical properties, and swelling behaviours of the photopolymerised and printed parts, a series of characterisation tests, including Fourier transform infrared spectroscopy (FTIR), differential scanning calorimetry (DSC), goniometry, tensile test, gel fraction measurement and pulsatile swelling study were performed on this study. In conclusion, the differences between polymerisation methods are significant; despite their chemical structures and thermal properties being similar, there were significant differences with regard to tensile properties, swellability and wettability of samples. The implications of conducting this study are remarkable, not only in providing a new way of preparing NVCL, but also in demonstrating the possibility of using 4D printed NVCL for practical applications.

## 1. Introduction

The three-dimensional printing (3D) technique, which is also known as additive fabrication and rapid prototyping, is a fast way to create objects based on pre-designed shapes and sizes. These processes may lead to the toolless production of finished goods and the mass production of individually customised parts [1]. Three-dimensional printing has slowly evolved to create one-of-a-kind devices. Everything from small machine components to large automotive and aerospace equipment can be created by 3D printing [2]. In the biomedical field, 3D printing technology can even be used to create therapeutic devices that fit individual patients, such as customised implants, diagnostic platforms and scaffolds for tissue engineering and drug delivery systems [3,4]. With the continuous development of science and technology, the 3D printer has evolved an ability to work with a variety of types of material, such as liquid resin, thermoplastic powder and filament. Each type of 3D printer employs different methods and principles to process the entity model.

Stereolithography (SLA) is a solid free formation technology and was first exposed to the public in the early 1970s by a Japanese researcher, Hideo Kodama [5]. SLA is a type of 3D printing technique which works by solidifying light-sensitive photocurable resin. Optical fabrication provides the capability to print a part with high accuracy [6]. An ultraviolet (UV) laser can solidify the photopolymer to create a link chain between molecules. The product is created layer by layer following the shape and size of a virtual model, which is drawn in the computer-aided design software (CAD) in advance [3]. Following the pre-designed model, the photopolymer resin is solidified by UV light to form a single layer onto the surface of the photopolymer vat; then, the elevator platform descends and repeats the process to form a new layer until the actual object is built from bottom to top. By removing the residual resin on the surface and the auxiliary layer used for building, the object is formed [7]. A decade ago, a top-down system process approach was also gradually applied to the technique of stereolithography. This method did not require a large amount of resin and created a smooth surface on the manufactured piece [6]. The solid freeform technique has been expertly applied in electronics components, jewellery manufacturing, and the automotive and biomedical industries [8,9,10]. The main benefit of the SLA technique is its capability to print a part with high resolution. When compared to the fused-deposition modelling (FDM) 3D printing, the support structures can be more easily removed. Also, nozzle jamming does not occur during the process [2]. In addition, due to the complex consolidation behavior and molecular diffusion of the selective laser sintering (SLS) process, SLA has a relatively wider choice of materials [11].

Poly (N-vinyl caprolactam) (PNVCL) is a temperature-responsive polymer. It is well-known for its exceptional biocompatibility, solubility, thermosensitivity, and non-ionic and non-toxic properties [12]. Furthermore, NVCL polymer exhibits a phase transition and becomes less water-soluble as the temperature increases above the lower critical solution temperature (LCST). The LCST of the homopolymer PNVCL (32–34 °C) is close to the physiological temperature [13], which offers great opportunities for the applications of targeted drug delivery carrier [14], matrix for microbial cell entrapment and cell culture scaffold [15]. In addition, the LCST behaviour of PNVCL is sensitive to changes in polymer concentration, molecular weight of the polymer and solution composition [16]. Henna et al. reported that, when compared with pure PNVCL, copolymer PNVCL-C_11_EO_42_, due to the CEO, offers more opportunities for hydrogen bond generation to create a more stable structure, which results in a steady and sustained drug release [17]. Free radical polymerisation is a well-established method for preparing PNVCL homopolymers and copolymers [14]. Its free radical synthesis has been performed in different media—bulk, toluene, benzene and water provide outstanding media for it [15].

By changing structures that can be converted in a pre-programmed manner in response to a stimulus, 3D printed materials can be modified to impart flexibility and boost utility. Four-dimensional printing was first initiated and labelled by a research group at MIT [18]. It is fundamentally the same as 3D printing; the difference is that the eventual states of the fabricated product can be changed again while a stimulus acts on it, such as temperature change, moisture change, pH change and magnetic change [19]. Since the development of 4D printing, most academics used shape memory polymers (SMPs) for achieving 4D printing, thanks to their plasticity and simple operability [10,20,21,22]. However, limited research has been conducted on the development of 4D parts using hydrogel-based NVCL copolymers. The works presented in our last paper [12] synthesised a series of PNVCL copolymers/terpolymers via photopolymerisation. At the same time, a new idea of achieving 4D printing by 3D printing of NVCL-based polymers was proposed. Based on the paper, the S5 samples containing 0.1 wt% Irgacure 2959, 2 wt% PEGDMA, 30 wt% DMAAm and 70 wt% NVCL demonstrated a short reaction time and excellent reversible swelling behaviour as the temperature rose and fell. These properties provide the possibility of practical 4D printing of S5 chemically crosslinked samples. Therefore, the main objective of this study is to use the candidate formulation to conduct 4D printing. This provides a new synthetic approach and additional application areas for NVCL. Additionally, this contribution compared the differences in material properties between the photopolymerised and 4D printed NVCL hydrogels, which provides a significant reference for how such NVCL gels can be practically applied in various fields. In this study, 4D printing was achieved by using a new highly photosensitive photoinitiator, H-Nu 400IL, and the differences between polymerisation methods and photoinitiator types are significant. The 4D printed jigsaws and flowers displayed intelligent and reversible expansion/shrinkage behaviour with increasing and decreasing temperature in aqueous media demonstrating the functionality and manipulability of the NVCL material.

## 2. Materials and Methods

### 2.1. Materials

N-vinyl caprolactam (NVCL) was obtained from Sigma Aldrich Ireland with a molecular weight of 139.19 g/mol and a storage temperature from 2 to 8 °C. The UV light-sensitive initiators, 4-(2hydroxyethoxy) phenyl-(2-hydroxy-2-propyl) ketone (Igracure^®^ 2959 Ciba Corp, New York, NY, USA), were obtained from Ciba Specialty Chemicals. H-Nu 400 IL is a broad wavelength liquid blend photoinitiator with easy addition to a free radical curable formulation. It was obtained from Spectra Photopolymers (Spectra Photopolymers Industry, Millbury, MA, USA). N,N-dimethyl acrylamide (DMAAm) was purchased from Sigma Aldrich (Wicklow, Ireland) with a molecular weight of 99.13 g/mol. The chemical crosslinker used was poly (ethylene glycol) dimethacrylate (PEGDMA) supplied by Sigma Aldrich (Wicklow, Ireland) with a molecular weight of 550 g/mol.

### 2.2. Patterns Design

Based on the previous study [12], the disc samples revealed excellent performance, displaying expansion and shrinkage behaviour as the temperature rose and fell. In order to explore whether further scenarios and patterns of 4D objects were able to display the same intelligent behaviour as disc samples, the complex three-dimensional objects were designed and prepared.

**Figure 1:**
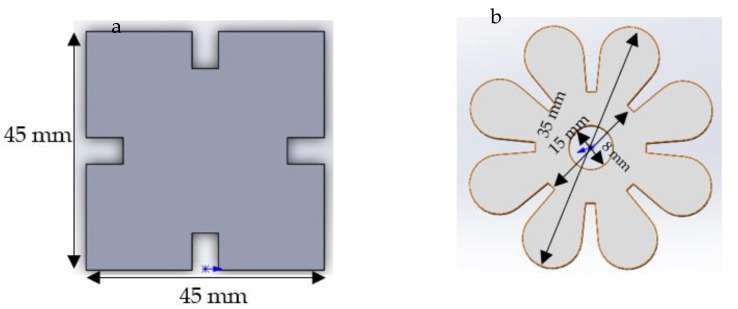
(**a**) Jigsaw and (**b**) flower shape models designed in Solidwork.

The model of a jigsaw and flower shape were designed in Solidworks software; the size and patterns are shown in Figure 1. The thickness of the models is 2 mm. In order to prepare the silicone mould for photopolymerisation, the jigsaw and flower prototypes were printed using the SLA Form 2 3D printer with V2 High Temperature resin (Formlab company, Berlin, Germany). The prototype made of High Temperature resin has tough properties to avoid adhesion during the mould processing.

### 2.3. Hydrogels Photopolymerisation

The hydrogels investigated in this study were prepared by free radical polymerisation using UV light. These hydrogels were synthesised using a UV curing system (Dr. Gröbel UV-Elektronik GmbH, Ettlingen, Germany). This particular irradiation chamber is a controlled radiation source with 20 UV-tubes that provides a spectral range between 315 and 400 nm at an average intensity of 10–13.5 mW/cm^2^. All samples were cured at high intensity (10–13.5 mW/cm^2^). The prepolymerised mixtures were prepared by combining the desired amounts of the monomer NVCL with specified amounts of other materials and photoinitiator and 2 wt% PEGDMA for chemical crosslinking gels. The batches were placed in a 100 mL beaker and mixed using a magnetic stirrer for 20 min until a homogeneous mixture was obtained. The solutions were pipetted into silicone moulds that contained disc, jigsaw and flower impressions. Photopolymerisation was carried out for 10 min on each side, and all samples were dried prior to use for 24 h in a vacuum oven at 50 °C. 

### 2.4. Form 2 SLA 3D Printer Parameter 

Form 2 (Formlab Company, Berlin, Germany) is a stereolithography 3D printer developed by Formlab. Items are constructed on the platform from bottom to top in an upside-down posture. Only files in STL format can be accepted and uploaded to the printer. Based on a model designed in advance in computer-aided design software (CAD), the photopolymer resin is solidified by providing UV laser (405 nm) to form a single layer onto the surface of the photopolymer vat; then, the elevator platform descends and repeats the process to form a new layer until the actual object is built. The laser spot size of the 3D printer is 140 microns and the maximum printing size is 14.5 × 14.5 × 17.5 cm^3^, with a range from 25 to 300 microns per layer. The thickness of print for each layer is set at 50 μm. A range of materials can be used for printing, including standard materials, tough and durable materials, flexible and elastic materials, rigid and structural materials, dental materials, biomedical materials, castable and specialty materials. The specific resin cartridge and tank and build platform should be correctly installed in the printer before the printing begins. During the printing process, the resin can be automatically filled into the self-heating resin tank. Depending on the material, the resin would be heated and kept at a constant temperature until the print is complete. Independently developed resins can also be used on this printer, but open mode needs to be activated. In this mode, the cartridge or tank detection function, resin heating, resin wiper, and resin dispensing are disabled. Additionally, the resin needs to be filled manually in the resin tank.

### 2.5. 4D Printing

In this study, 4D printing was achieved using the SLA 3D printer (Formlab, Form 2) to print homemade resin. The prototype is created layer-by-layer following the model designed in advance using computer-aided design software (CAD). Preform software is the specific app to upload the model to the printer. The orientation and numbers of the model can be adjusted in Preform software automatically or manually. The printer should be switched to open mode for third party resin use. After the model was uploaded to the printer, the homemade solution was prepared in the same way as the UV-cured hydrogels. Based on the formulations, the monomer was first placed into a 250 mL beaker and other solutions were added to the beaker using a pipette to prepare a mixture. The preparation of the H-Nu 400IL samples had to be performed in a dark environment. Therefore, aluminum foil was used to surround the beaker to avoid prepolymerisation. To achieve homogeneity, a magnetic stirrer was used to stir the mixture for 20 min at 50 °C heat. All in all, 150 g of solutions were prepared and poured directly into the resin tank in preparation for printing. After printing, the residue solution remaining on the printed objects was removed with a tissue paper containing IPA. The finished parts were placed into the UV box to post cure for 20 min. The formulations used for 4D printing are listed in Table 1. The profile [23] provided by Spectra Photopolymers Industry suggests testing several concentrations in a “ladder” study between 1–5 wt% to achieve the best results, and the unpresented works performed in our lab found that the 4D parts printed with a 2 wt% H-Nu 400IL exhibited the best quality. Therefore, the comparison of material properties was carried out on 2 wt% samples.

### 2.6. Attenuated Total Reflectance Fourier Transform Infrared Spectroscopy

Attenuated total reflectance Fourier transform infrared spectroscopy (ATR-FTIR) was carried out on a Perkin Elmer Spectrum One FT-IR Spectrometer (C-001), fitted with a universal ATR sampling accessory. All data were recorded at room temperature (<20 °C) in the spectral range of 4000–650 cm^−1^, utilising a 4-scan per sample cycle and a fixed universal compression force of 75 N. Subsequent analysis was performed using Spectrum software. The tests were performed in duplicate for each sample. 

### 2.7. Differential Scanning Calorimetry

The LCST of the chemically crosslinked hydrogels was detected by Differential Scanning Calorimetry (TA instrument 2920 Modulated DSC, New Castle, DE, USA). The DSC was calibrated with indium standards. The hydrogel samples were allowed to swell until equilibrium in distilled water at room temperature. The sample’s surface was wiped with moistened filter paper to remove free water and placed into the aluminum pans and weighed out, ranging from 8 to 12 mg, using a Sartorius scale with 0.01 mg resolution. DSC measurement was performed on swollen samples from 10 °C to 60 °C at a rate of 1 °C/min, referenced against an empty pan. All samples were examined under a pure nitrogen atmosphere at 30 mL/min. The results were plotted as a function of heat flow (W/g) against temperature (°C). The tests were performed in triplicate.

### 2.8. Goniometry

Contact angle goniometry is used to assess a solid substrate’s capacity to resist liquids [24]. The xerogels were placed onto the stage area and a dynamic sessile droplet of water was placed on the sample surface while simultaneously photographing the spread of the drop over the surface of the xerogel. The angle measurement can be automatically performed on the photo screen. In this study, photos at 0 s and 115 s were used to analyse the wettability of the xerogels. All tests were carried out in triplicate. 

### 2.9. Tensile Test

Tensile testing is a destructive test process that provides information about the tensile strength, yield strength and ductility of the materials. It measures the force required to break a composite or plastic specimen and the extent to which the specimen stretches or elongates up to that breaking point [25]. The tensile strength at break of samples was analysed in line with ASTM standard D 638 Type V, using a Lloyd LRX tensometer set up in tensile mode. Each sample was measured in five copies. Specimens were mounted and strained at a rate of 20 mm per minute until failure occurred.

### 2.10. Gel Fraction Measurement

Gel fraction measurement may be used as a quantitative indicator of the efficiency of hydrogel network formation [26]. The gel fraction of all batches was measured using a round disc. The xerogel samples were placed into covered Petri dishes of distilled water at room temperature until equilibrium swelling was achieved. Once equilibrium swelling was attained, samples were dried in the vacuum oven at 100 Pa and 50 °C until no change in weight was observed. Each sample was measured in three copies. Gel fraction (%) was calculated using Equation (1), where W_0_ is the weight of the xerogel after photopolymerisation, and W_d_ represents the dried weight of the sample after extraction of soluble parts.

Gel fraction (%) = W_d_/W_0_ × 100% (1)

### 2.11. Pulsatile Swelling Study

Initially, the apparent dry weight (W_0_) of the photopolymerised and printed samples was measured. The samples were placed into rectangular plastic containers of 120 × 80 cm^2^ containing 350 mL of distilled water (pH = 7.1). Samples were tested under switching conditions between ambient temperature and 50 °C. The plastic containers were first stored at room temperature and weighed after specific intervals (W_t_) to determine water uptake until the swelling equilibrium was reached. Then, these samples were placed into the oven to raise the temperature to 50 °C (above LCST) and the weight of the samples was measured again. After the new equilibrium state was reached, these plastic containers were restored to room temperature. Before each measurement, the excess distilled water was dried with filter paper. The measurement was conducted in three copies for each sample. The expanding percentage for each time was calculated using Equation (2).

Swelling Ratio (%) = (W_t_ − W_0_)/W_0_ × 100% (2)

where W_t_ and W_0_ are the weights of the gels in the swelling state at a predetermined time and the initial dry mass of the dried state, respectively.

### 2.12. Statistical Analysis

A statistical comparison of results was performed using a one-way ANOVA with Tukey post-tests to determine differences between each batch. Differences were considered significant when *p* < 0.05. The software package used to perform statistical analysis was IBM SPSS Statistics. The *p* values for each experiment are explained in the result figure legends.

## 3. Results and Discussion

### 3.1. The Preparation of Photopolymerisation and 4D Printing Samples

Both photopolymerisation and SLA 3D printers use UV light to cure the liquid-based materials. However, unlike photopolymerisation, the UV light provided by 3D printers is intermittent and only acts regularly on a pre-defined trajectory based on the pattern and shape of the product. Therefore, it is necessary to investigate the material property differences between the stereolithography and photopolymerisation.

**Figure 2:**
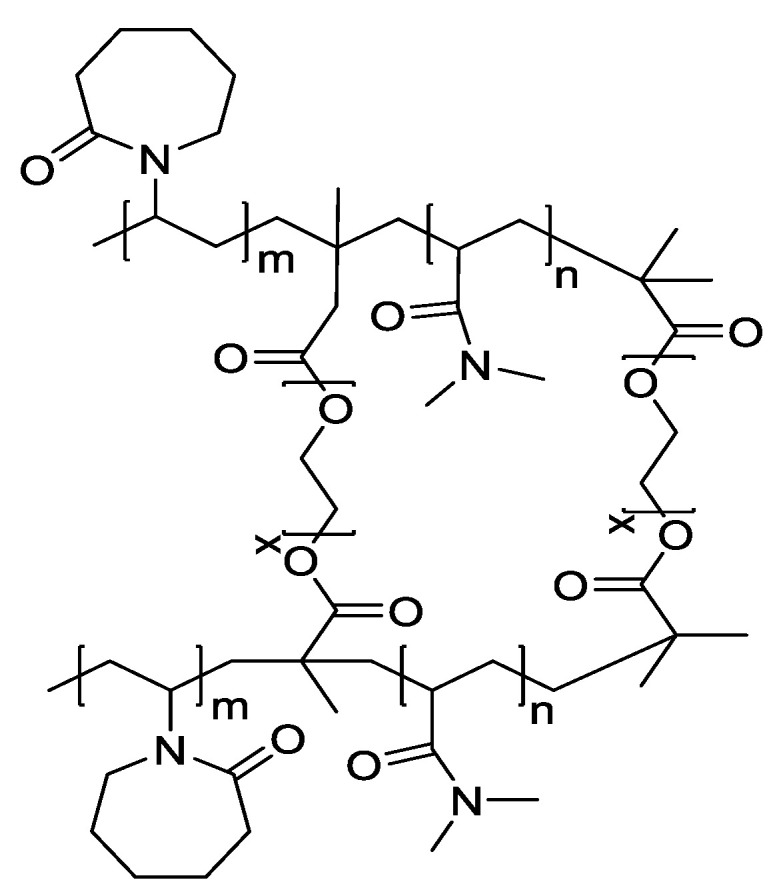
The chemical structure of P(NVCL-DMAAm).

**Figure 3:**
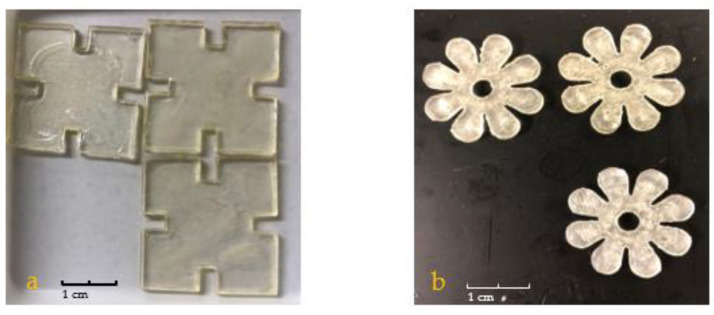
The appearances of the UV-cured (**a**) jigsaw and (**b**) flower samples.

Figure 2 shows the chemical structure of the P(NVCL-DMAAm) samples. For photopolymerisation, the jigsaw and flower samples were prepared based on U1 formulation. All samples were cured on a silicone moulding and dried for at least 24 h in a vacuum oven before use. Just as with the disc samples previously, the jigsaw and flower samples displayed a bright, shiny and transparent appearance. Nevertheless, all samples cured well and successfully formed the shape of the jigsaw and flower. This phenomenon indicates that the chains are arranged randomly and present no actual boundaries or discontinuities from which light can be reflected [27], thus indicating that the samples are amorphous and were successfully prepared. The appearances of the UV-cured jigsaw and flower samples are shown in Figure 3.

Based on the paper [12], the S5 samples (which are U1 in this study) exhibited the most suitable properties for 4D printing. Therefore, in this study, the 4D printing trials were performed on the U1 formulation first. For a common 3D printing process, a support structure is always built with the items. This gives better assurance of the quality and integrity of the product. However, considering the particular properties of the homemade resin, it was anticipated that it would be difficult to print the complex support structures. Therefore, the sample was designed to print without supports. The model of the flower and jigsaw was designed to be built horizontally. However, after three attempts, the homemade resin was unable to print. No solid parts were observed. In order to ensure the accuracy of the experiment, the resin used in the printer was pipetted in the silicone mould and an attempt was made to cure it in the Dr. Gröbel UV curing system. As a result, the resin can be fully cured via photopolymerisation. Comparing the differences between the two technologies, the reason why the homemade resin is unprintable may be that the laser scanning time for each layer is too short, so the new layer of resin cannot be cured on the basis of the previous layer. This may also be attributed to the fact that the intensity of the ultraviolet light provided by the printer is poor, resulting in the inability of the self-made resin to cure. Based on the failure analysis, a new photoinitiator, H-Nu 400IL, was selected that better matched the wavelength of the 3D printer and the dosage of the photoinitiator was increased. 

**Figure 4:**
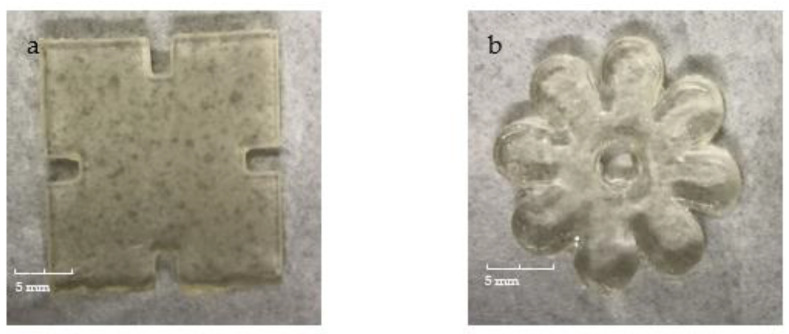
The appearances of 3D printed (**a**) jigsaw and (**b**) flower samples.

The second 4D printing trial was conducted using the new formulation P2. As a result, the prints could be built on the platform with an excellent appearance finish and could be easily removed from the platform by means of a small spatula for the post-curing process. The appearances of the printed parts are shown in Figure 4. As can be seen, the printed jigsaws and flowers exhibited high fidelity to the original design, and fully express the dimensions and patterns of the design. In addition, the surface of the prints is bright, smooth and flat. These performances all confirm the quality and accuracy of the printed parts. Therefore, these prints are eligible for further performances and fourth dimensional behaviours assessment.

### 3.2. Attenuated Total Reflectance Fourier Transform Infrared Spectroscopy

**Figure 5:**
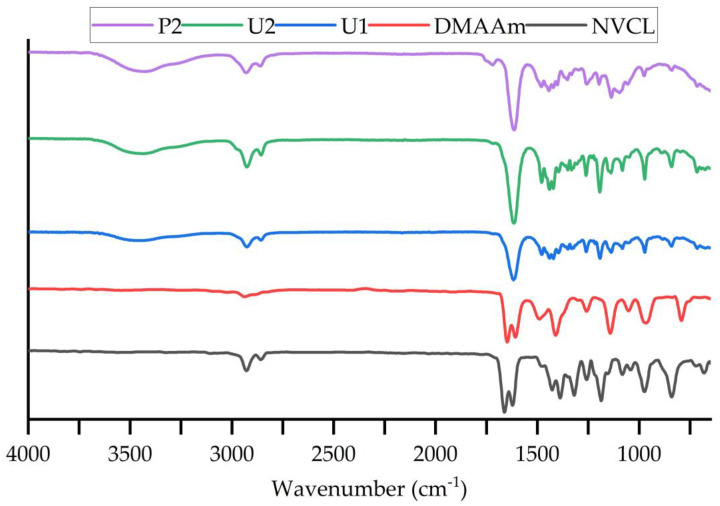
FTIR spectra of NVCL, DMAAm, U1, U2 and P2 samples. *n* = 2.

ATR-FTIR was used to detect the differences in the chemical structure between the photopolymerisation and 3D printing samples. When infrared radiation passes through the sample, some of the radiation is absorbed by the sample and some radiation is passed (i.e., is transmitted). The signal generated at the detector is a sample that represents the spectral “fingerprint" of the molecule. Different structures or chemical bonds can be distinguished following the spectral curve. Table 2 summarises the band value of each chemical bond that exists in the monomer and synthetic samples. The displayed characteristic absorption bands of the three samples largely matched. The transmittance of each material is shown as %T on the Y-axis. The monomers displayed characteristic absorption bands at 1622/1662 cm^−1^ (NVCL), and 1608/1648 cm^−1^ (DMAAm). These bands are assigned to the carbonyl group C=O bond stretching vibration and the C=C bond stretching vibration. In the P(NVCL/DMAAm) spectra, only an intense peak at 1618/1615/1614 cm^−1^ in U1, U2 and P1 samples is attributed to the characteristic absorption of C=O stretching. Based on the previous studies [12], only one peak exists at the wavelength between 1600~1700 cm^−1^ in the printed and cured sample spectra, which indicates successful polymerisation and printing of the samples (C=C bonds disappearance [28]). The bands at 1479 cm^−1^ (U1), 1479 cm^−1^ (U2) and 1481 cm^−1^ (P1) are attributed to the stretching of C-N bonds in both monomers [29]. The O-H bending arising at 3442 cm^−1^ and 3425 cm^−1^ in photopolymerised and printed samples provides further evidence that the polymerisation and 3D printing have succeeded [30]. As can be seen in Figure 5, it is worth noting that some of the peaks detected in the printed samples were lower or higher in intensity than the two photopolymerised samples. This suggests that the number of chemical bonds created in the printed samples may be slightly different compared with those created in the photocured samples [31]. For example, photopolymerisation will produce more C=O bonds than the 3D printing process. However, the water content in the printed sample is higher than that in the sample formed by photopolymerisation. Compared to light-cured samples, 4D printing is time consuming. As the part is built up layer by layer, the sample being printed and the solution are more exposed to moisture in the air, resulting in high water content.

### 3.3. Differential Scanning Calorimetry

**Figure 6:**
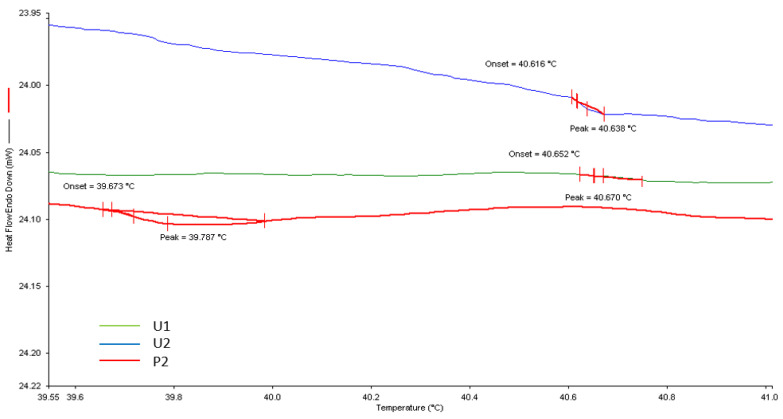
A thermogram illustrating the LCST of UV-cured and printed P(NVCL-DMAAm) samples. *n* = 3, *p* > 0.05.

Differential scanning calorimetry is a thermal analytical technique used to measure a material’s heat capacity, its endothermic and exothermic transitions, and its relationship to change in temperature [32]. The LCST of those photopolymerised and printed chemically crosslinked P(NVCL/DMAAm) were detected by DSC. The gel samples were immersed in distilled water at room temperature and allowed to swell to equilibrium before the DSC measurement. The onset point of the endothermal peak was defined as the LCST. As can be seen in Figure 6, the DSC profiles show that the LCSTs of U1, U2 and P2 printed are 40.7 °C, 40.6 °C and 39.7 °C, respectively. There was no significant difference between polymerisation methods and photoinitiator types. As the temperature increased, all the samples exhibited the same LCSTs at around 40 °C. This is because the chemical composition of the U1, U2 and P2 hydrogels were identical. The same hydrophilic/hydrophobic balance in the polymer side chains determines the particle-incorporated gels’ similar phase separation behaviour [33].

### 3.4. Goniometry

The contact angle provided the level of the hydrophobicity held by each hydrogel. The hydrophobicity of the UV-cured and printed hydrogels was determined using goniometry. The wettability of the sample surface is measured by measuring the contact angle between the water droplet and the dry xerogel surface. The more the liquid spreads across the solid surface of the material, the more attracted to the material it is. The closer the contact angle is to 0°, the greater the hydrophilicity of the sample material. Materials with a contact angle up to 90° are considered hydrophilic, those with contact angle greater than 90° are considered hydrophobic, and surfaces with contact angle greater than 150° are considered to be superhydrophobic, as the liquid droplet meets the surface without significant wetting [34].

**Figure 7:**
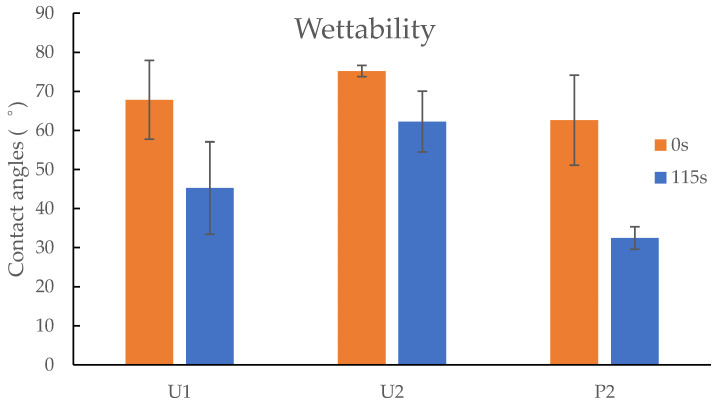
The comparison of contact angles between U1, U2 and P2 samples. *n* = 3, *p* > 0.05 (0 s), *p* < 0.05 (115 s).

As demonstrated in Figure 7, the U1 copolymer P(NVCL-DMAAm) prepared by 0.1 wt% Irgacure 2959 has a mean contact angle of 67.8° at zero seconds, dispersing into a drop with a mean contact angle of 45.3°, characterising it as a hydrophilic material. The same trend was observed in U2 samples, which have a mean contact angle of 75.2° at zero seconds, spreading into a drop with a mean contact angle of 62.3° at 115 s. However, the P2 samples prepared via 3D printer have a mean contact angle of 62.6° at zero seconds, dispersing into a drop with a mean contact angle of 32.5° at 115 s. Based on the wettability measurements, SLA significantly increases the hydrophilicity of P(NVCL-DMAAm) compared to UV chamber samples. This coincides with the FTIR findings, where the printed samples show stronger peaks of O-H bonds than the UV polymerised samples. Furthermore, when comparing the two UV-cured samples, the Irgacure 2959 sample contact water angle was closer to 0°, which meant that the mix of H-Nu 400IL reduced the hydrophilicity of P(NVCL-DMAAm) compared to the Irgacure 2959 blend.

### 3.5. Tensile Test

**Figure 8:**
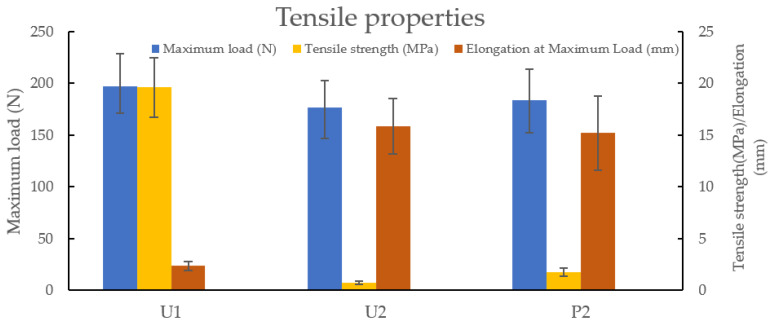
The comparison of tensile properties between U1, U2 and P2 samples. *n* = 5, *p* > 0.05 (maximum load), *p* < 0.05 (tensile strength), *p* < 0.05 (elongation).

The tensile test is used to find out how strong a material is and also how much it can be stretched before breaking. The mechanical strength can be assessed by the appearance before and after water absorption. However, the behaviour of NVCL in terms of changes in mechanical properties after blending with other polymers can be more precisely analysed through tensile testing. The tensile tests were performed on the U1, U2 and P2 samples to investigate the differences between maximum load, tensile strength and elongation at maximum load. As shown in Figure 8, there was no significant difference in the maximum load for each sample. U1 samples are slightly stronger than both U2 and P2 samples. The same trend has also been found in terms of tensile strength, where U1 UV-cured samples have a greater tensile strength at limit. This result could be due to the samples initiated by Irgacure 2959 being less plastic than those prepared with H-Nu 400IL, resulting in a greater tensile strength. In the absence of plasticity, the samples tend to be brittle. This conclusion was approved by the results of elongation at maximum load; the samples manufactured with H-Nu 400IL are more ductile than those manufactured with Irgacure 2959. However, there are no relevant contributions reported with similar results. In conclusion, tensile properties are significantly influenced by the type of photoinitiators, but the polymerisation method does not affect them.

### 3.6. Gel Fraction Measurement

**Figure 9:**
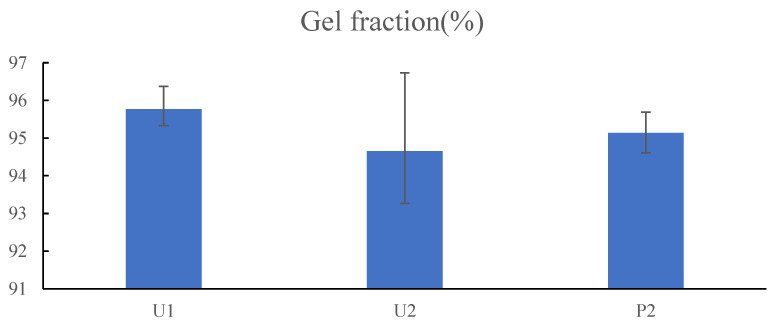
The comparison of gel fraction between U1, U2 and P2 samples. *n* = 3, *p* > 0.05.

Gel fraction can be used as a qualitative indicator of the efficiency of network formation [35]. The greater the gel fraction percentage, the greater the covalent bonding. Gel fraction is the mass fraction of the network material resulting from a network forming polymerisation or crosslinking process. The improved stability of the hydrogels is attributed to the denser crosslink structure of the copolymer network. Figure 9 shows the gel fraction percentages of P(NVCL-DMAAm) hydrogels prepared by different photoinitiators and polymerisation methods. The result is that the different photoinitiators and polymerisation methods have no effect on the gel fraction. This can be explained by the fact that the polymerisation method and the photoinitiator have no influence on the chemical structure of the hydrogels. Thus, three hydrogels of P(NVCL-DMAAm) were able to maintain a similar gel fraction of about 95%. Only 5% weight loss confirms the excellent quality of the cured and printed parts.

### 3.7. Pulsatile Swelling Study

**Figure 10:**
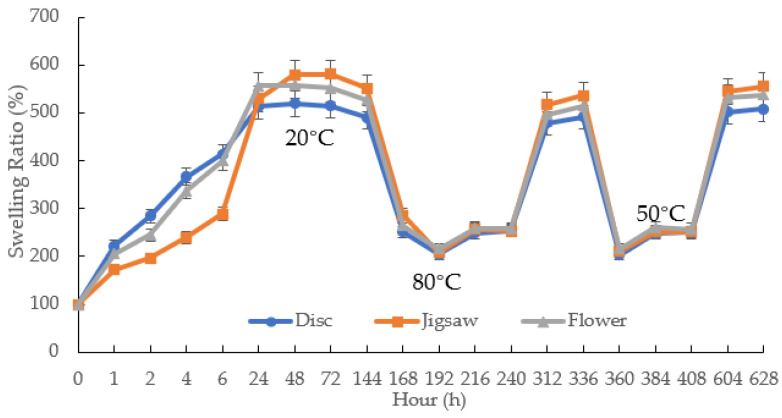
The pulsatile swelling behaviours of photocured chemically crosslinked P(70NVCL/30DMAAm) copolymer hydrogels with different shapes. *n* = 3.

**Figure 11:**
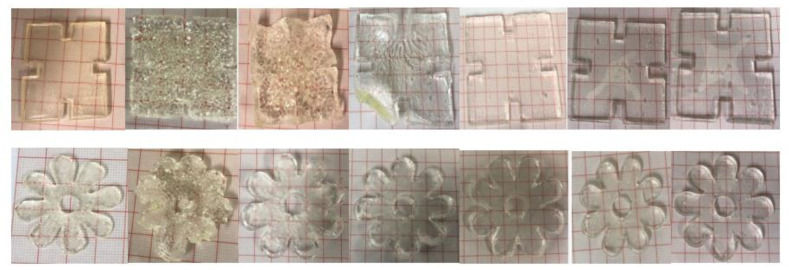
The appearance of the photopolymerised jigsaw and flower samples at times (from left): 0 h; 2 h; 4 h; 6 h; 72 h; 216 h; 336 h.

The pulsatile swelling tests were carried out on both photocured and printed samples to further examine the smart 4D behaviours of the shaped samples. Taking into account the potential expanding dimensions of the samples, swelling analysis of samples was performed in a rectangular plastic container of 120 × 80 cm^2^. Based on the U1 formulation, the first part of this study aimed to investigate the swelling behaviour differences between the discs and jigsaw- and flower-shaped samples. As can be seen in Figure 10, the curves of the shaped samples and discs are fundamentally consistent. At temperatures below LCST, all samples reach their maximum swelling capacity in water and retain their structural integrity completely. Nevertheless, for all P(70NVCL-30DMAAm) samples, they can expand up to five times the weight of the original xerogels. It is worth noting that the disc and flower samples can reach equilibrium after 24 h, while the jigsaw samples take 48 h to reach their maximum capacity. This phenomenon can be explained by the slow contact of the internal structure with water due to the large dimensions of the jigsaw samples. After the temperature is raised to above the LCST, the polymers shrink to an equilibrium state after 24 h. Moreover, when the temperature continuously rises to 80 °C, the formed jigsaw and flower samples are able to continuously deswell and reach a new equilibrium level. This performance reveals the same trend as chemically crosslinked discs. When the temperature was dropped back to 20 °C, all samples were able to return to their swollen state. The appearances of the jigsaw and flower sample during the swelling test are shown at Figure 11 (the appearances of the disc sample during the swelling studies can be found in paper [12]). Consistent with the disc samples, the appearance of the shaped samples exhibited a bright, transparent appearance throughout the pulsatile swelling test. The flower and jigsaw shapes can be perfectly represented, with a smooth surface and well-defined edges after expansion and contraction. This indicates that cyclable intelligent expansion behaviour can be successfully achieved by complex shape designs.

**Figure 12:**
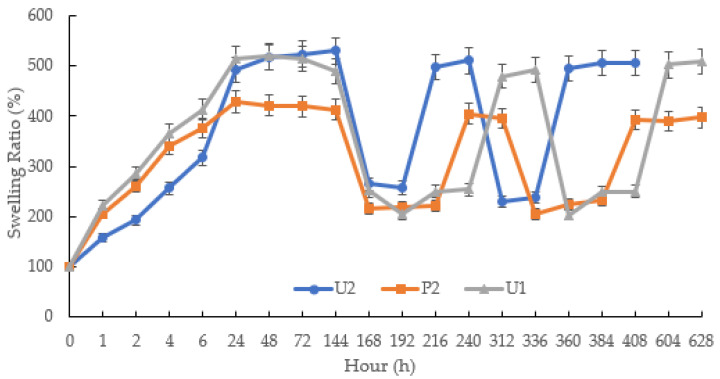
The pulsatile swelling behaviours of photocured and printed chemically crosslinked P(70NVCL/30DMAAm) copolymer hydrogels. *n* = 3.

The aim of the second part pulsatile swelling study was to compare the differences in dimensional expansion/shrinkage capabilities between the photopolymerised and printed samples. Comparative tests were carried out on the jigsaw and flower samples. As demonstrated in Figure 12, the U1, U2 and P2 samples were able to exhibit reversible shape change behaviour as the temperature rose and fell. At temperatures below LCST, it was observed that the maximum swollen weight had been reached after 24 h. Those items that were photocured (U1, U2 sample) can expand up to five times the weight of the original xerogels, while the printed parts only reached an expansion ratio of 400%. This could be due to the high water content of the prints. When the temperature was above LCST, the samples began to shrink; the expansion of the shrunken samples dropped to 200% for both printed and cured parts. In the subsequent cycles, the expansion ratios of the photocured and printed samples were consistent with the results obtained in the first pulse experiment.

**Figure 13:**
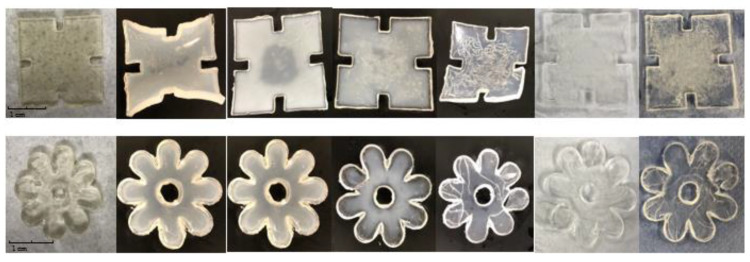
The appearance of the 4D printed jigsaw and flower samples sample at times (from left): 0 h; 6 h; 24 h; 72 h; 168 h; 240 h; 408 h.

The appearances of 3D printed jigsaw and flower samples are shown in Figure 13; both the swollen flower and jigsaw shapes are able to maintain structure integrity and display the complete appearance in the swelling/deswelling cycles. However, unlike the photocured parts, the prints changed from transparent to white during the water absorption process. After the first equilibrium state is reached, both the flower and the jigsaw shape are completely white.

In summary, the jigsaw flower samples manufactured via UV chamber system displayed the same reversible expansion/contraction properties as the disc samples. The quality of the swollen parts can be assured. A smooth surface and excellent appearance can be maintained. However, samples with large dimensions require a longer time to reach their maximum capacity. Additionally, 4D printing has been successfully achieved in this study using P2 formulation. These prints were able to demonstrate intelligent and reversible expansion/shrinkage behaviours. In comparison, for the photopolymerised samples, the response time for both the UV-cured and printed parts was 24 h. However, the UV-cured samples exhibited a stronger swelling capacity than those prints.

On the whole, by using a new photoinitiator, 4D printing was successfully achieved using an NVCL-based polymer based on the formulation S7 developed in the previous article [12]. The 4D prints prepared with the formulations containing 2 wt% H-Nu 400IL, 2 wt% PEGDMA, 30 wt% DMAAm and 70% wt% NVCL exhibited a perfect appearance and were able to respond to temperature pulses across their critical temperatures. In addition, Table 3 shows the effect on the material properties of NVCL samples prepared via different means of polymerisation methods and photoinitiator types has been examined. As a result, both photopolymerised and printed shaped samples show gel fraction around 95%. The UV-cured and printed P(70NVCL/30DMAAm) parts were able to demonstrate expected expansion/shrinkage behaviour in water media. Moreover, when viewing the chemical structures and lower critical solution temperatures, there was no significant difference between those items. Nevertheless, based on the contact angle measurements, SLA significantly increased the hydrophilicity of NVCL compared to UV chamber samples, and the samples prepared by Irgacure 2959 were more hydrophilic than those samples made by H-Nu 400IL. As for the tensile properties, U1 is more brittle and slightly stronger than both U2 and cured P2 samples.

In this study, the potential functions of the material NVCL have been discovered. The printed and UV-cured samples can be used as a size changeable demonstrator. Additionally, by using this method, some of the more complex original 3D models could be created and become a new type of dimensional changeable toy in the future. However, the volume-changing behaviour exhibited by the hydrogel material still seems extremely homogenous compared to most other 4D printing reports.

Therefore, it would be an advancement in future research if some other types of materials could be combined with hydrogel-based polymers through a multi-material 3D printer to achieve a wider variety of shape transformation behaviours. By incorporating with flexible or rigid materials, the deformation generation on the hydrogel can spur the soft material change, leading to a change in dimensions and curvature. According to this principle, a series of products such as grippers [36,37], actuators [38,39,40], load carriers [41] and electronic components [9] could be produced. In addition, by controlling the amount, proportion, combination and position of the two materials, specific shape-shifting behaviour such as self-curving, self-bending, self-twisting and self-helixing can be achieved, which allows this combo to be used in more complex structures or devices, thus demonstrating its greater value.

## 4. Conclusions

In contrast to other homemade 4D printable resins prepared in the literature, this contribution exploits a unique material and rapid preparation method suitable for 4D printing. In this study, 4D printing was successfully achieved by using a temperature-responsive polymer made from NVCL. Using the Form 2 SLA 3D printer, the 4D printed jigsaws and flowers were perfectly printed and were able to show intelligent and reversible swelling/deswelling behaviour in water media after the temperature rose and fell. By carrying out FTIR, DSC, goniometry, tensile test, gel fraction measurement and pulsatile swelling studies, the effects on material properties of NVCL samples prepared via stereolithography and UV chamber polymerisation were also investigated. Overall, the differences between polymerisation methods and photoinitiator types are significant; although their chemical structures and thermal properties are similar, there were significant differences with regard to tensile properties, swellability and wettability of samples. Based on the findings of this study, the practical 4D printing concept was successfully achieved using the NVCL-based polymer. This offers not only the possibility of using NVCL in a new field distinct from biomedical applications, but also offers a fresh material for 4D printing research.

## Figures and Tables

**Table 1:** The formulations of photopolymerisation and 4D printing samples.

Sample Codes	Photoinitiators		Monomers		Crosslinker	Polymerisation Methods
	Irgacure 2959	H-Nu 400 IL	NVCL	DMAAm	PEGDMA	
	(wt%) (365 nm)	(wt%) (300~430 nm)	(wt%)	(wt%)	(wt%)	
U1(S5) P1	0.1	--	70	30	2	UV Curing
	0.1	--	70	30	2	3D printing
U2 P2	--	2	70	30	2	UV Curing
	--	2	70	30	2	3D printing

**Table 2:** FTIR bands for constituent monomers and UV-cured and 3D printed samples.

Functional Group					
	NVCL	DMAAm	U1	U2	P2
Aliphatic C-H	2929, 2858	2937	2927, 2857	2926, 2857	2926, 2858
C=O	1622	1608	1618	1615	1614
C-N	1480	1490	1479	1479	1481
-CH_2_-	1428	1424	1422	1422	1422
C=C	1662	1648	---	---	---
=CH and =CH_2_	3105, 975	969	---	---	---
O-H	---	---	3442	3442	3426

**Table 3:** The material properties comparison between different photoinitiators and polymerisation methods.

Material Properties	Photoinitiators	Polymerisation Methods
Chemical structure (FTIR)	Negligible	Negligible
Thermal properties (DSC)	Negligible	Negligible
Wettability (Goniometry)	Significant	Significant
Tensile properties	Significant	Negligible
Gel fraction	Negligible	Negligible
Swellability (Pulsatile swelling)	Significant	Significant

## Data Availability

Not applicable.
